# The metaplastic mosaic of Barrett’s oesophagus

**DOI:** 10.1007/s00428-018-2317-1

**Published:** 2018-03-03

**Authors:** Sujata Biswas, Michael Quante, Simon Leedham, Marnix Jansen

**Affiliations:** 1 Nuffield Department of Medicine, University of Oxford, Oxford, UK; 2 II. Medizinische Klinik, Klinikum Rechts der Isar, Technische Universität München, Munich, Germany; 3 UCL Cancer Institute, London, UK; 4 University College London Hospital, London, UK

**Keywords:** Metaplasia, Barrett’s oesophagus, Inflammation, Lineage tracing

## Abstract

Barrett’s oesophagus surveillance biopsies represent a significant share of the daily workload for a busy histopathology department. Given the emphasis on endoscopic detection and dysplasia grading, it is easy to forget that the benefits of these screening programs remain unproven. The majority of patients are at low risk of progression to oesophageal adenocarcinoma, and periodic surveillance of these patients is burdensome and costly. Here, we investigate the parallels in the development of Barrett’s oesophagus and other scenarios of wound healing in the intestine. There is now increased recognition of the full range of glandular phenotypes that can be found in patients’ surveillance biopsies, and emerging evidence suggests parallel pathways to oesophageal adenocarcinoma. Greater understanding of the conditions that favour progression to cancer in the distal oesophagus will allow us to focus resources on patients at increased risk.

## Introduction

“It is hard to make a patient without complaints feel better.”

Oesophageal adenocarcinoma surveillance (‘Barrett’s screening’) encapsulates some of the best and some of the worst that early cancer detection programs have to offer. The initiation of these surveillance programs has been prompted by the increase in oesophageal adenocarcinoma incidence over the last 40 years [1, 2]. Although squamous cell carcinoma remains the predominant malignant condition affecting the oesophagus in many developing nations around the globe, in the west, oesophageal adenocarcinoma has increased by about 5% annually since the 1970s [3]. The majority of oesophageal adenocarcinoma patients still present at advanced stages of disease, and overall 5-year survival consequently remains poor at around 15%. Given these figures, there is a clear and imminent need for diagnostic tools that will help us find patients at increased risk.

Barrett’s oesophagus (BO) is the most proximate risk factor for oesophageal adenocarcinoma development. The condition is defined as the metaplastic replacement of squamous epithelium with columnar epithelium in the distal oesophagus in response to acid-biliary reflux. Importantly, the increase in oesophageal adenocarcinoma incidence is paralleled by an apparent rise in BO prevalence, further supporting the concept that BO is the key precursor lesion to oesophageal adenocarcinoma [4]. However, determining the exact population prevalence of BO is less straightforward than it may seem at first. This is principally because heartburn complaints are common in the general population and there is only a weak association between the reported presence of heartburn and the development of BO [5]. An inherited predisposition plays some part in Barrett’s development as indicated by GWAS studies that compared Barrett’s patients and normal controls. These studies have revealed that BO occurs more commonly in individuals carrying one (or more) of a small set of genetic risk polymorphisms [6]. This suggests that an inherited predisposition can determine the tissue response to acid-biliary reflux. Intriguingly, some of these polymorphisms are found in genes involved in oesophageal embryonic development. This illustrates the complex multifactorial aetiology of Barrett’s development, wherein an environmental factor precipitates the development of a precursor lesion in individuals carrying genetic risk alleles.

A further factor that complicates the assessment of the true population prevalence of BO is the simple fact that the formal definition of the condition varies between nations. The European Society for Gastrointestinal Endoscopy (ESGE) and American Gastroenterology Association (AGA) guidelines both require that intestinal metaplasia be documented on oesophageal biopsy, because intestinal metaplasia is seen as a sine qua non for cancer progression [7]. By contrast, the British Society for Gastroenterology (BSG) guidelines stipulate that BO is primarily an endoscopic diagnosis [8]. Although this difference may seem trivial, in practice, this may mean that a given patient is eligible for Barrett’s surveillance in London, whereas this same patient, after relocating to Amsterdam, would no longer be eligible for surveillance.

For these reasons, population estimates of BO vary between 1 and 5% [9]. Even if the true estimate is closer to the lower bound, then there is still a large reservoir of individuals at some risk of oesophageal adenocarcinoma. However, despite the aforementioned *relative* annual increase in oesophageal adenocarcinoma incidence, the *absolute* risk remains low and retrospective population-based studies have demonstrated a progression rate of around 0.10–0.13% per patient per year [10, 11]. In other words, within a random sample of 50,000 individuals, around 500 of these individuals will have BO (1% prevalence), and of these 500 individuals, only 1 will develop oesophageal adenocarcinoma in a given year. These figures indicate that the majority of patients with BO have a long-term benign condition. In fact, because of shared co-morbidities (male gender, obesity), the absolute risk of dying of cardiovascular disease is much greater than the risk of dying of oesophageal adenocarcinoma in patients with BO [12]. Even if we put important considerations of finite resource allocation and healthcare spending aside, given these numbers, we should carefully consider whether inclusion of patients in Barrett’s surveillance programs does indeed strike a fair balance between the protection afforded by early endoscopic diagnosis and the risks and negative quality-of-life impact of cancer screening [13].

## Lack of evidence in favour of Barrett’s surveillance

What then is the evidence that Barrett’s surveillance reduces oesophageal cancer mortality? Surprisingly, the evidence for this is thin at best. In fact, some studies have concluded that there is no evidence that Barrett’s surveillance reduces cancer mortality. For example, in a case-control study carried out by the group of Doug Corley on the efficacy of Barrett’s surveillance endoscopy, the authors showed that there was no association between exposure to upper gastro-intestinal endoscopy and oesophageal cancer death [14]. Indeed, patients who had died from oesophageal adenocarcinoma were just as likely to have undergone surveillance endoscopy as control patients. Now, because this was a community-based study, there were many confounders the authors could not control for. However, the absence of *any* protective effect of endoscopy is stunning. The evidence in favour of surveillance endoscopy comes mainly from case-control studies wherein Barrett’s patients included in screening programs were compared with appropriate controls. As might be expected, inclusion in a screening program correlates with detection of cancer at earlier stages, which is less likely to have spread beyond the oesophagus, and this translates to longer overall survival times for patients who were diagnosed with oesophageal adenocarcinoma whilst on a surveillance program [15]. However, these uncontrolled studies are susceptible to many biases, including length-time and lead-time biases.

In short, there is no direct evidence that Barrett’s surveillance on balance reduces oesophageal cancer mortality. Much of this uncertainty likely relates to the fact that BO remains an underdiagnosed condition. Indeed, most patients with newly diagnosed oesophageal adenocarcinoma have no history of either BO or heartburn complaints [3]. The clinical return and cost-benefit to society of Barrett’s surveillance have, for all of these reasons, come under increased scrutiny. It is hoped that trials such as the British BOSS (Barrett’s Oesophagus Surveillance Study) and the German BarrettNET studies, wherein a total of over 5000 Barrett’s patients will be followed for over 10 years to investigate all-cause and disease-specific mortality between patients who have been randomly allocated to two-year endoscopic surveillance or conservative clinical follow-up, will finally provide us with guidance on this issue. However, the trial’s sample size and length of follow-up are a clear indication of the complexity and magnitude of this endeavour.

Pathologists play a significant role in this debate over the efficacy and efficiency of Barrett’s surveillance. A significant share of the daily workload for a busy department can consist of oesophageal screening biopsies. At the same time, pressures on the pathology workforce have never been higher with staffing levels not increasing at the same level as the demand [16]. Given the minimal reported benefit of current surveillance practices for oesophageal adenocarcinoma, it is clear that we must find ways that allow patient selection with greater precision and at reduced cost [17, 18].

## The Barrett’s metaplastic mosaic

The seemingly straightforward histopathologic definition of BO as a metaplastic condition whereby the native squamous epithelium of the distal oesophagus is replaced with columnar epithelium belies an altogether far more complex microscopic picture. In fact, rather than a single phenotype, patients’ biopsies show a range of columnar phenotypes. It is important to stress that despite the traditional emphasis on the cellular composition of the epithelium per se, the Barrett’s segment is organised, like all mucosal layers of the gastro-intestinal tract, into a quasi-repetitive arrangement of glands. Every gland is maintained by a unique population of stem cells, and every gland is therefore a singularly evolving unit within the mucosal sheet [19]. These metaplastic glandular units come in a variety of appearances, but, importantly, (1) the morphology of the various types of glandular units is invariant within and between patients, and (2) the morphology of these metaplastic gland types often mimics gland types found elsewhere in the gastro-intestinal tract, either natively or in pathologic conditions. With these ground rules in mind, we will first cover the various gland phenotypes found in the mature Barrett’s segment, before discussing what we know regarding the dynamics of these gland phenotypes.

**Fig. 1:**
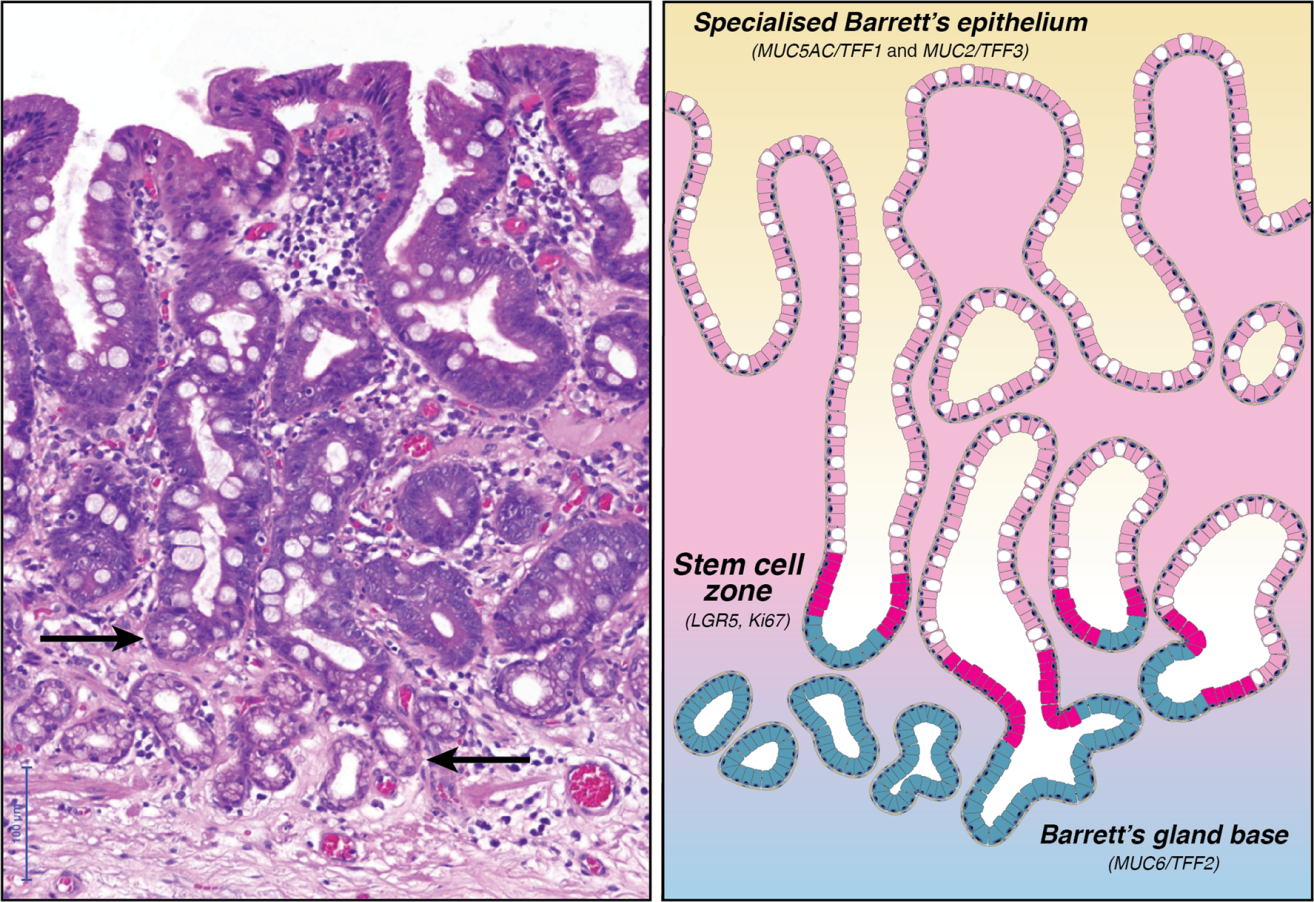
The canonical Barrett’s gland. Overview of the canonical Barrett’s gland, left H&E and right cartoon. Details are given in the text. The Barrett’s gland demonstrates bidirectional flux from a stem cell compartment about one third up from the base of the gland. The mucous base produces bicarbonate and MUC6/TFF2 (marked by arrows), whilst the superficial crypt compartment is lined by goblet cells (marked by MUC2/TFF3) and foveolar cells (marked by MUC5AC and TFF1) (Reprinted with permission from [21])

The gland phenotype canonically associated with BO displays a mixed epithelial lining consisting of scattered goblet cells against a background of columnar cells with mucinous properties indistinguishable from gastric foveolar cells [20] (Fig. 1). This dual mixed gastric and intestinal patterns of epithelial differentiation are reflected in its mucin core peptide and trefoil factor (TFF) expression pattern with goblet cells producing the intestinal type mucin MUC2 as well as trefoil factor 3 (TFF3), whilst foveolar cells produce the gastric type mucin MUC5AC and trefoil factor 1 (TFF1) [22]. These MUC proteins contain abundant oligosaccharide side-chains, which allow these proteins to bind copious amounts of water after secretion into the gut lumen. These MUC proteins further self-aggregate, which creates a thick visco-elastic gel that coats the underlying epithelium [23]. This peculiar pattern of mixed gastric and intestinal lineage differentiations has been widely described as ‘specialised epithelium’ or ‘specialised metaplasia’, or, in the vernacular of pathology reports, ‘intestinal metaplasia’. Note that in less recent publications, this may have been referred to as type II or type III incomplete intestinal metaplasia; however, this histochemical subtyping is now considered obsolete [24].

Important to this discussion is the functional organisation of this canonical Barrett’s gland. Unlike crypts in the small intestine and the colon, where stem cells reside strictly at the base of the gland and move up along the crypt (and villus) as these cells differentiate and mature, in Barrett’s glands, the stem cell compartment is located about one third up the height of the gland, and mature cell lineages show a bidirectional flow from this stem cell compartment both towards the lumen as well as towards the base of the gland. This was shown in an experiment wherein oesophageal adenocarcinoma patients scheduled for oesophageal resection were infused with a thymidine analogue (IdU) at varying time points before surgery [22]. Tracing the distribution of this indelible label in daughter cell populations confirmed bidirectional migration within these Barrett’s glands and showed that cellular migration towards the glandular base compartment of the gland occurred much slower than towards the superficial crypt compartment of the gland; whereas the label had been all but lost from the superficial crypt population in little over a week, non-dividing IdU-positive cells were detected up to 10 weeks after label infusion in the gland base population. This mucous base of the Barrett’s gland is lined by a population of columnar cells that produce not only MUC6, but also bicarbonate (HCO3^−^). This buffers the caustic refluxate and, together with the mucinous gel blanketing the metaplastic mucosa, protects the lining of the distal oesophagus. This functional compartmentalisation is relevant to a complete understanding of the unique functional properties of the canonical Barrett’s gland [21].

Expression of the LGR5 stem cell marker about one third up the height of the gland further supports this as the location of the stem cell niche [22]. LGR5 mRNA is localised at the junction of the MUC5AC+/TFF1+ cells and the MUC6+/TFF2+ cells, the origin of the bidirectional cell flux and site of maximum proliferative activity (as shown by Ki-67 immunohistochemistry). These observations, thus, provide an initial framework for understanding the cellular makeup and functional properties of the canonical Barrett’s gland. Note that this bidirectional compartmentalization is by no means unique in the gastro-intestinal tract and strongly resembles the basic architecture of the pyloric gland in the gastric antrum.

**Fig. 2:**
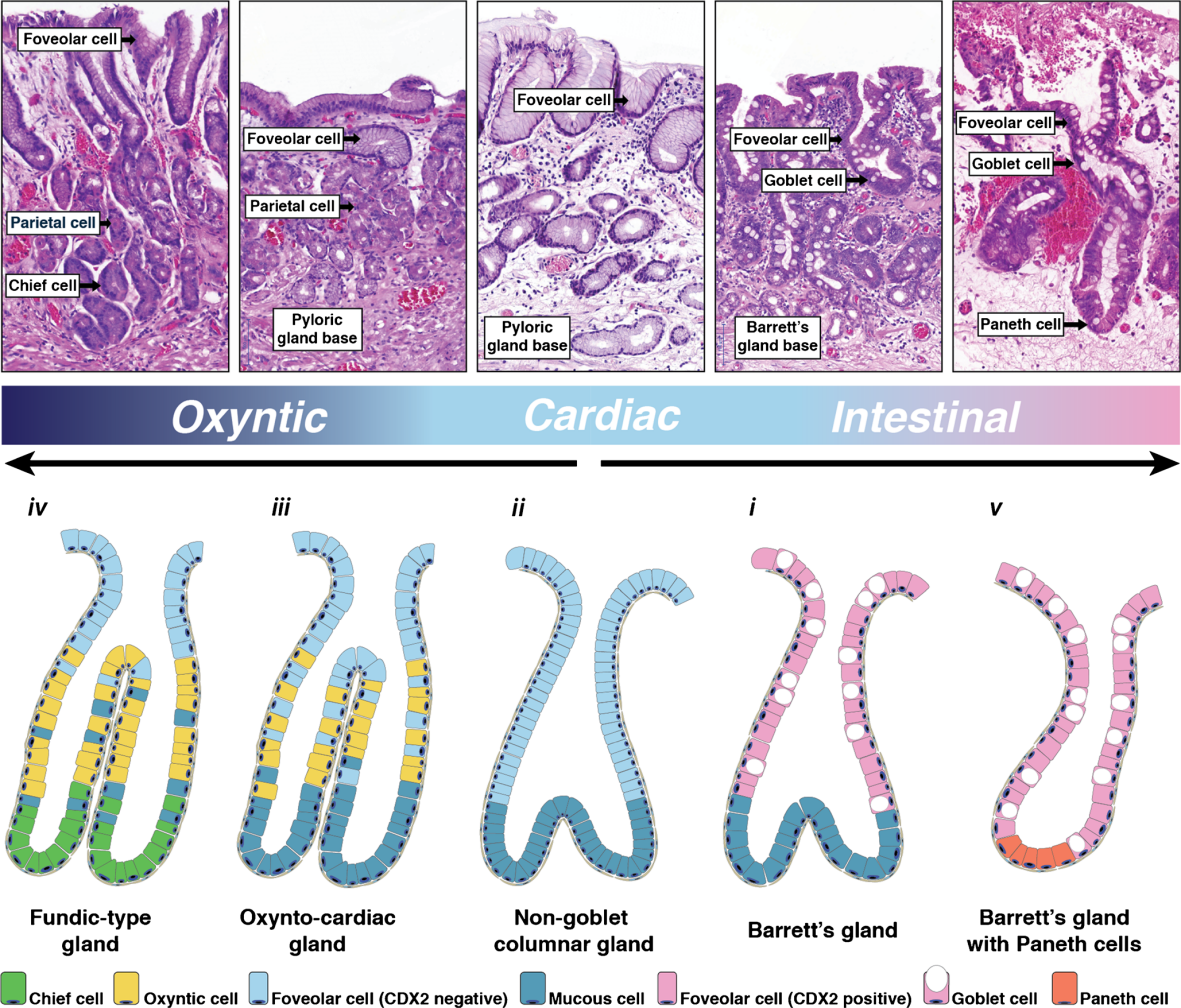
Gland phenotypes in the metaplastic mosaic of Barrett’s oesophagus. Barrett’s biopsies show a defined spectrum of glandular phenotypes ranging from oxyntic glands, comparable to those in the corpus of the stomach, to glands with mature intestinal differentiation with enterocytes and Paneth cells. These latter glands are indistinguishable from complete intestinal metaplasia in the human stomach. Other gland types (oxynto-cardiac glands, simple mucous glands and canonical Barrett’s glands as in Fig. 1) fall in between these extremes. Together, these five gland phenotypes represent five stages along the spectrum from oxyntic gastric to intestinal differentiation (Reprinted with permission from [21])

There are a small number of other gland types, which together constitute the metaplastic mosaic of the columnar oesophagus (Fig. 2). The best studied of these is the cardiac gland (also transitional gland or non-goblet columnar gland). In essence, the epithelial lining and bidirectional architecture of the cardiac-type gland are identical to those of the canonical Barrett’s gland described above, save for an absence of goblet cells in these glands. This makes these glands the simplest, in terms of differentiated epithelial cell types, of all the Barrett’s gland types, showing only MUC5AC/TFF1 foveolar cells along the superficial crypt compartment and MUC6/TFF2 cells along the mucous base. These glands have been quite extensively studied, as this is the gland type most commonly found in biopsies of patients with short-segment BO and in biopsies from patients with columnar metaplasia of the neodistal oesophagus following oesophageal resection. Together, this suggests that this gland type may be the earliest recognisable (‘early responder’) gland type in developing BO. It is also a gland that is indistinguishable in terms of glandular architecture and cellular makeup from reparative glands known as pseudo-pyloric metaplasia found in, for example, terminal ileitis in Crohn’s disease.

The remaining gland types are variations on a theme. The cardiac-type gland may show oxyntic differentiation in the form of scattered parietal cells at which point the gland is essentially comparable to similar glands found in the transitional mucosa of the gastric incisura or gastric pylorus (Note that contrary to common belief, parietal cells are not restricted to corpus-type mucosa and are abundantly found in normal human pyloric mucosa [25].). Mature chief cells may also be found in these glands, which show the complete complement of cell types normally found in the gastric body and fundus, although the irregular packing of these glands clearly indicates that this is non-native, post-inflammatory mucosa. Indeed, in many cases, these glands can be found in the context of unambiguous landmarks of the anatomic oesophagus such as submucosal gland complexes, strongly suggesting that these fundic-type glands developed as part of the metaplastic mosaic. Whether these metaplastic oxyntic glands develop from cardiac glands and, if so, whether these represent true stem cell-derived metaplasias or are a manifestation of variable levels of oxyntic gland differentiation are important areas of future research, since these suggest malleable levels of differentiation of these archetypal gastric glands with important implications for the temporal dynamics of glandular metaplasia in the atrophic stomach.

Finally, glands showing mature intestinal differentiation may be seen, which show Paneth cells at the base of the gland and enterocytes along the superficial crypt compartment. These are the only glands completely lacking gastric mucin core proteins and are also the rarest of all the gland types described above, although patients with abundant Paneth cell differentiation may occasionally be seen.

## What is the origin of Barrett’s cancer?

The phenotype of Barrett’s mucosa is therefore truly protean, consisting of a range of glandular phenotypes of intestinal and gastric differentiations. Whether intestinal differentiation is required for tumour progression has long remained a matter of debate [8]. Over the years, a great deal of evidence has been generated to support either position. For example, epidemiological studies employing histopathology registry data have suggested that the cancer progression rate is higher in patients with intestinal metaplasia on oesophageal biopsy than in patients without goblet cells [10], although other epidemiological studies have failed to find evidence for this claim [26]. Yet other groups have directly examined the rate of molecular abnormalities in non-dysplastic Barrett’s epithelium with and without intestinal metaplasia and reported that the rate of molecular alterations is greater in intestinal metaplasia than in non-intestinalised epithelium. Again, however, independent studies have failed to confirm this [27, 28]. In an effort to trace the origins of Barrett’s adenocarcinoma, Kaiyo Takubo and co-workers examined the adjacent epithelium in a series of minute Barrett’s cancers, some as small as 3 mm [29]. He showed that most of these lesions were surrounded by non-intestinalised epithelium strongly suggesting that these cancers could originate without direct intestinalised precursor. Critics of this carefully executed study retorted that Takubo had not examined the entire specimen and that some cancers may have simply obliterated their intestinalised precursors. In a follow-up study, Junko Aida examined a new series of minute cancers, this time meticulously sectioning at regular intervals through the entire specimen [30]. The authors essentially replicated the original findings, although, obviously, this could not refute the first point of critique that these minute cancers may have obliterated its metaplastic precursor lesion.

**Fig. 3:**
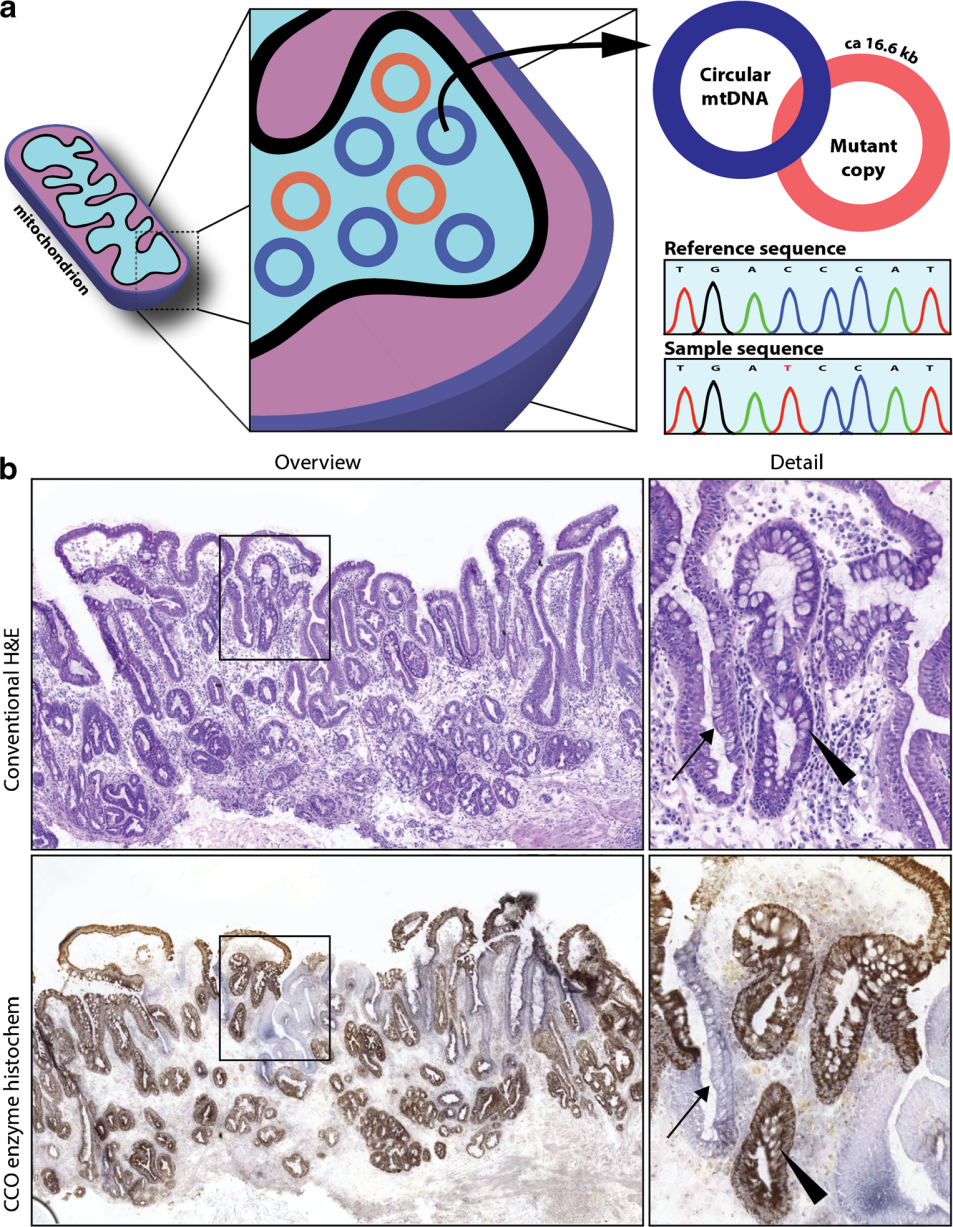
Lineage tracing through mitochrondrial CCO mutations. **a** CCO lineage tracing makes use of somatic mutations which occur in the mitochondrial respiratory chain. The respiratory chain maintains the electrochemical proton gradient across the inner mitochrondrial membrane. Somatic mutations in the mitochondrial DNA (mtDNA) may functionally inactivate proteins of the electron transport chain. This results in the loss of substrate conversion in the enzyme histochemical cytochrome *c* oxidase reaction. mtDNA is circular; wild-type copies are shown as blue rings whilst mutant copies are shown as red rings. Through drift, the number of mutant copies can increase within the cell and this can be detected by sequencing the mtDNA genome. **b** Example of CCO lineage tracing on snapfrozen Barrett’s mucosa. The top panel shows an H&E-stained consecutive section as a reference. A clone is detected which displays the loss of CCO substrate conversion in blue (lower panel). Wild-type epithelium retains substrate conversion enzyme activity and is labelled brown (DAB substrate precipitation). Notice how the clone expands within the mucosa. This epithelial clone lacks intestinal differentiation and was also shown to carry a mono-allelic p53 mutation (see also [31]). Arrows indicate CCO-deficient epithelium, and arrowheads indicate CCO-proficient epithelium

We recently provided the first direct evidence for oesophageal adenocarcinoma derivation from non-intestinalised epithelium [31]. To this end, we snap-froze a complete strip of Barrett’s mucosa running from the squamocolumnar junction to the proximal stomach as a Swiss roll. This strip was obtained from a resection specimen showing a small nodular Barrett’s adenocarcinoma at the squamocolumnar junction. By retracing the ancestry of the malignant clone to the neighbouring metaplastic epithelium using CCO lineage tracing (explained in Fig. 3), we found that this cancer actually originated in metaplastic cardia-type epithelium, which indeed lacked goblet cells and CDX2 expression. The non-dysplastic cardia epithelium shared a number of pathogenic mutations with the nodular cancer, including a mono-allelic TP53 mutation. This last result provides unambiguous evidence that pathogenic TP53 mutations can accumulate and clonally expand in cardia-type epithelium.

These data were obtained from a single patient, and further evidence will require follow-up work in a larger cohort. However, this result unequivocally shows that intestinal metaplasia is not the sole precursor to cancer in the metaplastic distal oesophagus. In fact, recent studies have suggested that progression to cancer may occur less often in patients with higher goblet cell counts [32, 33]. This suggests that goblet cell differentiation may in fact *protect* against neoplastic transformation. Together, these lines of evidence point to parallel lines of progression in the distal oesophagus. Work carried out by the group of Gregory Lauwers has revealed that many early neoplastic Barrett’s lesions can be separated, based on their expression of aforementioned gastric (MUC5AC, MUC6) and intestinal markers (CDX2, MUC2 and CD10), into either gastric- or intestinal-type dysplasia [34]. Whether these differences indeed reflect separate molecular pathways to oesophageal adenocarcinoma and, importantly, whether these dysplasias have a different clinical outcome will require further study. In conclusion, evidence is now slowly building that cancer progression in BO is not restricted to the intestinal lineage.

## Phenotypic dynamics of Barrett’s across time and space

Above, we have seen that Barrett’s mucosa is not a single epithelial entity but actually comprises a mosaic of glandular phenotypes, both intestinal and gastric in nature. Barrett’s biopsy specimens commonly show a diverse admixture of these phenotypes. Remarkably, the distribution of these gland phenotypes appears to show recurrent patterns, both in time and in spatial localization along the Barrett’s segment. Spatially, intestinalised phenotypes are found more proximal at the squamocolumnar junction, whereas cardiac and oxyntocardiac gland phenotypes are proportionally more common around the gastro-oesophageal junction [35–37]. For example, Harrison and co-workers found that intestinal differentiation was almost twice as commonly found in proximal biopsies near the squamocolumnar junction when compared to the most distal biopsies taken around the GOJ with a clear stepwise gradient in between [35]. It appears that the density of glands containing intestinal differentiation correlates with the pH gradient along the distal oesophagus; the less acidic the local average pH (i.e. closer to the squamocolumnar junction), the higher the proportion of glands with goblet cell differentiation. The significance of this spatial distribution of gland phenotypes is currently unclear. We have suggested that this distribution in gland types may be a reflection of local selection for phenotypes most adept at surviving in the harsh Malthusian environment of the acid-biliary refluxate-exposed distal oesophagus [21]. The central driver of this local ecology may be the soluble component of bile acids, which act as a detergent and solubilise lipid cell membranes through micelle formation. Classic studies on the solubility of bile salts show that it depends on luminal pH. Bile salt solubility is the greatest at intermediate pH ranges seen most proximally, whereas bile acids are insoluble and therefore incapable of forming micelles at pH ranges in the distal oesophagus [38]. Relatedly, in vitro studies have shown that solubilised duodenal bile salts are a strong inducer of CDX2 expression and goblet cell differentiation [39, 40]. The pH gradient along the Barrett’s segment may thus in turn set up a proximo-distal gradient of bile solubility, and this may explain the relative proportion of specific gland phenotypes along the length of the oesophagus.

Temporal analysis of gland phenotype distribution is complicated by the simple fact that in most patients the Barrett’s segment remains completely static over time and does not expand (or contract) despite years, or in some cases decades, of endoscopic follow-up, even in the context of ongoing oesophageal exposure to acid-biliary reflux [41]. However, a select population wherein columnar metaplasia of the (remnant) distal oesophagus does develop de novo has patients that have undergone cardia-oesophagectomy because of oesophageal cancer. In these patients, normal sphincter function is lost which may provoke gastro-oesophageal reflux, and consequently, in about half of these patients, columnar mucosa develops in the remnant distal oesophagus. Longitudinal studies show that the length of columnar mucosa increases over time, and histopathologic analysis demonstrates that the glandular phenotype changes over time from purely cardiac-type mucosa to canonical Barrett’s glands with intestinal differentiation [42]. These observations suggest that the earliest glandular phenotype that develops in the reflux-damaged distal oesophagus is the simple cardiac-type gland, which may evolve over time to show intestinalisation or further gastric differentiation. Supporting this contention are studies which show that some cardiac-type glands may show early intestinalisation with submaximal expression of Villin and CDX2 [43]. Muc2 is generally only expressed when goblet cells are morphologically evident. Finally, we have recently shown the clonal ancestry of canonical specialised Barrett’s glands and non-intestinalised cardiac-type glands, showing that indeed, these various phenotypes do not develop independently but are the result of phenotypic variation within glands that derive from a common ancestor [31].

This observation of a temporal phenotypic progression from cardiac-type glands to intestinalised epithelium is recapitulated in a recent mouse model of BO [44]. In this transgenic mouse model, IL-1beta is overexpressed in the oesophageal and squamous forestomach epithelia. The transgenic mice first exhibited spontaneous oesophagitis and then progressed to Barrett metaplasia at the gastro-oesophageal junction. Indeed, the addition of bile acids to the drinking water (0.2% deoxycholic acid) accelerated the onset of intestinal metaplasia. Taken together, these data strongly suggest that the earliest morphologic manifestation of glandular differentiation in the distal oesophagus is the simple cardiac-type gland, which may evolve over time to show either specialised intestinal differentiation or specialised gastric oxyntic and chief cell differentiation.

## What is the tissue origin of Barrett’s oesophagus?

What if we ask what drives the columnar transformation of the distal oesophagus when acid-biliary reflux first hits the naïve squamous mucosa of the distal oesophagus? This is a question of great contention and one on which opinion is sharply divided. Several models have been proposed so far, but the two models that have (arguably) been studied the most are a direct squamous-to-columnar transdifferentiation model and a repeated-wounding model with expansion of glandular progenitors from the proximal cardia. The transdifferentiation model proposes that squamous stem cells chronically exposed to the corrosive effects of acid-biliary reflux slowly change their differentiation lineage by downregulating the native squamous expression program in favour of a columnar cell expression program through the upregulation of lineage-determining factors such as SOX9 [39, 40]. Recent support for this model comes from a trial wherein reflux patients successfully treated with proton pump inhibitors were asked to discontinue acid suppression for 2 weeks [45]. Even within this relatively short timeframe, all patients showed progressive complaints, some with severe erosive (Los Angeles grade C) reflux oesophagitis, indicating that this is a pertinent model to study this disease. Biopsies from non-eroded areas showed severe squamous lymphocytic infiltration, suggesting that these inflammatory events mediate important aetiologic events.

**Fig. 4:**
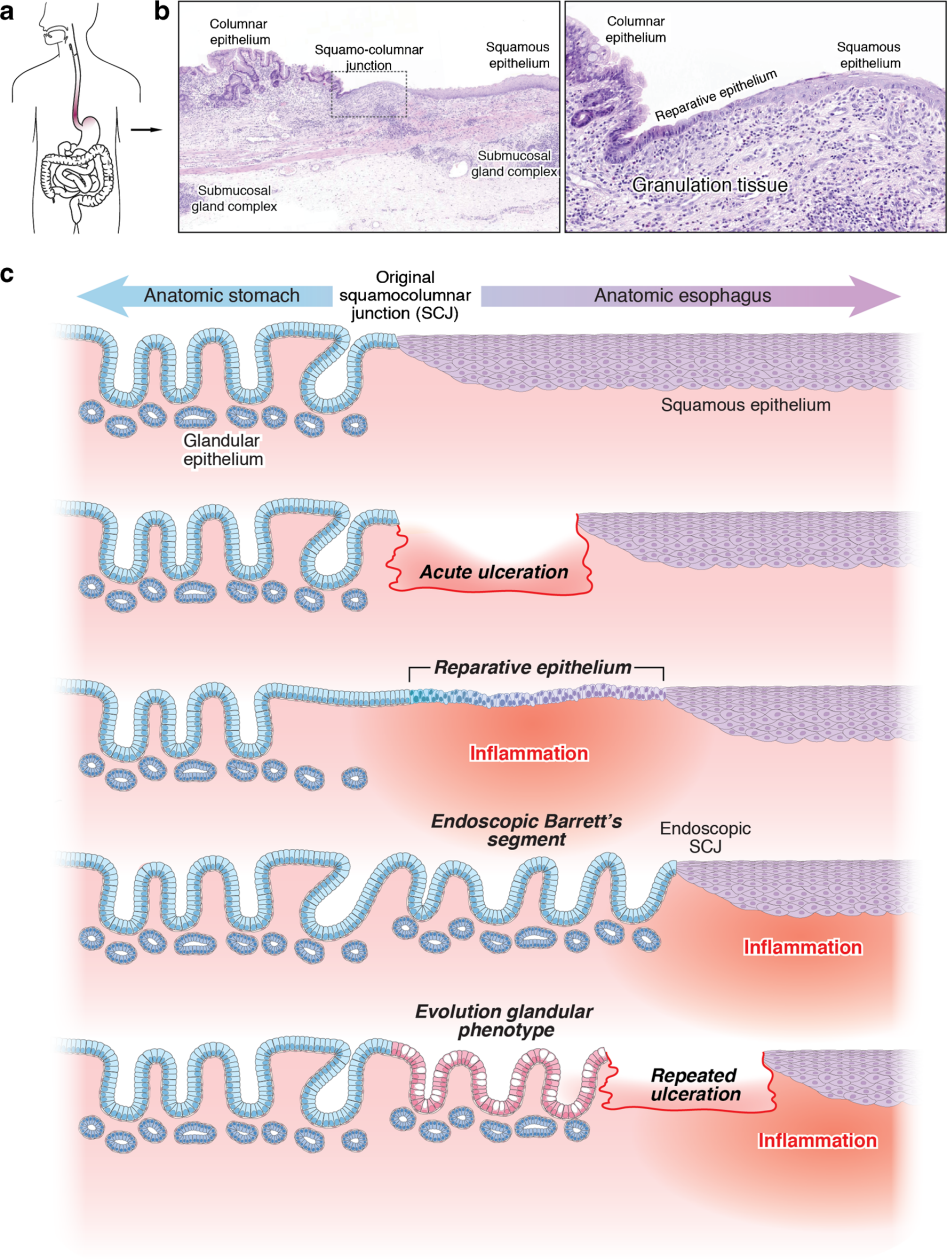
Chronic wounding and repair drive Barrett’s development. **a** Overview of a Barrett’s segment in the distal oesophagus. **b** Squamocolumnar junction in an oesophageal resection specimen shows a mono-layer of undifferentiated epithelium. **c** Expansion of Barrett’s mucosa through repeated cycles of wounding and repair (details in the main text) (Reprinted with permission from [46])

On the other hand, the chronic-wounding model proposes that with continuous micro-trauma due to caustic reflux, small patches of squamous epithelium are eroded, which are filled in through a normal wound-healing process (Fig. 4). According to this model, the local inflammatory wounding response drives proliferation of nearby progenitors to cover the epithelial defect. If wounding occurs at the squamocolumnar junction, then this will elicit proliferation of adjacent squamous and columnar progenitors. Although this is essentially a stereotypical wound-healing response, the reflux micro-environment will drive secondary selection for phenotypes best adapted to this ecology thus favouring mucin-producing columnar progenitors. With recurrent bouts of reflux and ulceration, the columnar epithelium expands, progressively replacing the distal oesophageal squamous epithelium.

Whether cyclic wounding and expansion occur within a short timeframe or progresses over many months or years is currently unclear. It may well be that progressive widening of the lower oesophageal sphincter due to slowly increasing abdominal pressure and increased reflux indeed progressively erodes the distal oesophageal epithelium over many years. There is some (indirect) evidence for this in patient studies [47]. For example, in carefully executed studies analysing oesophageal reflux in patients using detailed pH and manometry measurements across the lower oesophageal sphincter, McColl and co-workers found that in patients with a large waist circumference, acid reflux extended more proximally in the lower oesophageal sphincter and, importantly, this correlated with a wider zone of cardiac-type columnar mucosa. Indeed, and somewhat anecdotally, it appears that in rare patients, the histologic squamocolumnar junction can actually move *distally*, covering the gastric cardia. James Going and co-workers describe exactly this phenomenon in a patient with longstanding atrophic gastritis in the context of pernicious anaemia who underwent gastrectomy because of an incipient gastric cancer. The resection specimen clearly showed a collar of squamous epithelium, which occupied the anatomic cardia [48]. Given the atrophic gastritis, the most parsimonious explanation is that in this case, due to continuous erosion of the friable gastric mucosa, the squamous epithelium outcompeted native glands in the proximal stomach.

## Wound healing and ecologic adaptation

Chronic mucosal erosion and wound healing in response to micro-trauma are central to the model for Barrett’s development discussed above. What is, in some ways, unique about Barrett’s is that it occurs at a junction of epithelial phenotypes. Similar competitive processes can be seen at other epithelial junctions such as the anorectal junction. Mucosal trauma is the common denominator of a wide range of clinical conditions ranging from inflammatory bowel disease (IBD), to NSAID-provoked ulceration, and *Clostridium difficile* infection. Although the inciting agent is different in every case (and in some of these cases, such as IBD, remains fundamentally unknown), the wound-healing response that follows mucosal ulceration occurs in a stereotypical manner. As in any epithelial tissue, the first phase of mucosal wound healing following ulceration is the formation of granulation tissue and the deposition of a small film of fibrinous exudate to protect the underlying tissues. This granulation tissue is then covered by an initial layer of undifferentiated reparative epithelium, which lacks functional properties such as mucin differentiation. The next phase is specific to the glandular epithelium of the gastro-intestinal tract and involves the lateral ingrowth of adjacent glands through gland budding or fission [49]. Gland fission occurs until the mucosal defect has been functionally repaired, leaving a mucosal ‘scar’ in the form of gland distortion and fibrosis.

Although the data from patients can be informative with regards to the various contexts wherein mucosal wound repair plays a key role, functional dissection of these processes is best done in animal models. Like homeostatic stem cell regulation, wound repair is also an evolutionarily conserved process. Most recently, models have also been established which allow repeated observation through an endoscopic biopsy-wounding protocol. Miyoshi created wounds in mouse colon with 1-mm^2^ endoscopic biopsies, which allowed clear time points to visualise existing crypts repairing the defect [50]. Wnt5a, a non-canonical Wnt ligand, was one factor required for restoration of homeostasis. After mucosal injury, a flattened layer of non-proliferative epithelial cells (wound-associated epithelium) emanated from crypts adjacent to the wound and migrated over the wound bed. Next, crypts adjacent to the wound formed lateral, open extensions like channels towards the centre of the wound bed. These resembled crypts but had a proliferative, undifferentiated cell population, suggesting areas of new crypt formation. Wnt5a-positive stromal cells were found near these wound channels in the area of mucosal injury. Wnt5a-positive cells were also located adjacent to non-proliferative wound channel epithelial cells. Wnt5a-positive mesenchymal cells may therefore induce new crypt formation by potentiating TGF-β signalling and locally inhibiting proliferation of the stem/progenitor cell population within the wound channel. These animal studies reaffirm the central role for the gland as a unit of tissue homeostasis in the gastro-intestinal tract. In Barrett’s, these processes occur within a micro-environment which allows the selective expansion of glandular epithelial progenitors. Lineage-tracing studies will be essential in documenting the origin of these glandular epithelial progenitors.

## Conclusion

Here, we have dealt mostly with normal tissue homeostasis in the hostile acid-biliary reflux environment of BO. The diversity of glandular phenotypes represents a rich untapped source of information, which may reveal important clues about Barrett’s histogenesis. Unravelling the principal environmental drivers of glandular expansion will require detailed studies on tissue morphology *and* ecology (e.g. bile acid concentration, pH manometry) along the segment. Chemoprevention studies in BO have suggested that the inflammatory environment can have a key impact on neoplastic evolution [51–53]. Understanding the ecological conditions that drive malignant evolution in Barrett’s may help us avoid burdensome and costly surveillance in patients at low risk of transformation [17] and intervene in patients at increased risk [54].
